# Down-expression of *TaPIN1s* Increases the Tiller Number and Grain Yield in Wheat

**DOI:** 10.1186/s12870-021-03217-w

**Published:** 2021-09-30

**Authors:** Fu Quan Yao, Xiao Hui Li, He Wang, Yu Ning Song, Zhong Qing Li, Xing Guo Li, Xin-Qi Gao, Xian Sheng Zhang, Xiao Min Bie

**Affiliations:** State Key Laboratory of Crop Biology, College of Life Sciences, Shandong Agricultural University, Tai’an, 271018 Shandong China

**Keywords:** *TaPIN1* genes, Tiller number, Grain yield, Wheat

## Abstract

**Background:**

Tiller number is a factor determining panicle number and grain yield in wheat (*Triticum aestivum*). Auxin plays an important role in the regulation of branch production. PIN-FORMED 1 (PIN1), an auxin efflux carrier, plays a role in the regulation of tiller number in rice (*Oryza sativa*); however, little is known on the roles of PIN1 in wheat.

**Results:**

Nine homologs of *TaPIN1* genes were identified in wheat, of which *TaPIN1-6* genes showed higher expression in the stem apex and young leaf in wheat, and the TaPIN1-6a protein was localized in the plasma membrane. The down-expression of *TaPIN1s* increased the tiller number in *TaPIN1-RNA* interference (*TaPIN1-RNAi*) transgenic wheat plants, indicating that auxin might mediate the axillary bud production. By contrast, the spikelet number, grain number per panicle, and the 1000-grain weight were decreased in the *TaPIN1-RNAi* transgenic wheat plants compared with those in the wild type. In summary, a reduction of *TaPIN1s* expression increased the tiller number and grain yield per plant of wheat.

**Conclusions:**

Phylogenetic analysis and protein structure of nine TaPIN1 proteins were analyzed, and subcellular localization of TaPIN1-6a was located in the plasma membrane. Knock-down expression of *TaPIN1* genes increased the tiller number of transgenic wheat lines. Our study suggests that *TaPIN1s* is required for the regulation of grain yield in wheat.

## Background

Tiller number of wheat (*Triticum aestivum*) is an important agronomic trait that contributes to grain production. Plant hormones, environmental signals, and genetic factors are involved in the regulation of tiller number [1–4]. Auxin is a hormone with polar transport characteristics, and its concentration gradient establishment is necessary for plant morphogenesis. Based on previous reports, shoot branching is correlated with polar auxin transport in pea (*Pisum sativum*) [5] and *Arabidopsis* [6, 7].

PIN protein is specific to auxin transport and is a limiting factor of auxin polar transport [8–10]. In *Arabidopsis*, eight PIN proteins are identified, which possess two conserved domains formed by transmembrane helices and a conserved central hydrophilic loop [11].

The initiation and growth of lateral branches (called tillers in grasses) are important factors in determining plant architecture and yield [12–14]. Previous studies have demonstrated that decreased auxin transport affects branching in the monocot plants, such as rice (*Oryza sativa*), maize (*Zea mays*), and switchgrass (*Panicum virgatum*) [15–17]. Auxin maxima created by PIN1 at the meristem surface are responsible for organ initiation [18, 19]. Twelve *ZmPIN* genes and two *PIN*-like genes in maize [20] and twelve *OsPIN* genes in rice had been identified [21]. Transgenic plants with a reduction of *OsPIN1* gene expression display an increase in tiller number and till angle, which provides a new insight into the functions of the *PIN1* family in rice [21]. Maize BARREN INFLORESCENCE2 (BIF2) regulates auxin transport through direct regulation of ZmPIN1a during maize inflorescence development [22]. In severe alleles of *bif2* maize mutant, ZmPIN1a and ZmPIN1b protein expression patterns and localizations are altered in the tassel and ear [20]. The *bif2* mutants form a needle-like inflorescence structure and lack branches, spikelet pairs, and florets in male tassels and female ears, which is similar to the phenotype of *pin1* mutant in *Arabidopsis* [16].

Wheat is an important food crop; however, *PIN1* genes and their functions in wheat are rarely reported. In this study, we identified nine *TaPIN1* genes in wheat and analyzed their expression patterns. Furthermore, we found that down-expression of *TaPIN1s* in wheat resulted in more tillers and grain yield of each plant. Our results suggest that *TaPIN1s* play important roles in the regulation of tiller number and grain yield in wheat.

## Results

### Identification of TaPIN1 genes in wheat

We used AtPIN1 protein sequence as query to blastp against wheat *EnsemblPlants* database to identify the homologs of *AtPIN1* gene in wheat. Sequence alignment revealed that wheat A, B, and D subgenomes have several homologs, including *TaPIN1-6a*, *TaPIN1-6b1*, *TaPIN1-6b2*, *TaPIN1-6b3*, *TaPIN1-6b4*, *TaPIN1-6d*, *TaPIN1-7a*, *TaPIN1-7b*, and *TaPIN1-7d*. Among these sequences, chromosome 6A has one copy and it was located in 543,394,469–543,397,613, chromosome 6B four copies and they were located in 593,711,770–593,714,658, chromosome 6D one copy which was located in 397,082,345–397,084,736, chromosome 7A one copy located in 148,414,887–148,418,041, chromosome 7B one copy located in 109,702,167–109,705,332, and chromosome 7D one copy located in 146,862,702–146,865,831. The sequences encoded putative products of 588, 521, 589, 595, 587, 589, 587, 586, and 586 amino acids, respectively.

### Phylogenetic analysis and predicted protein structure of TaPIN1s

**Fig. 1:**
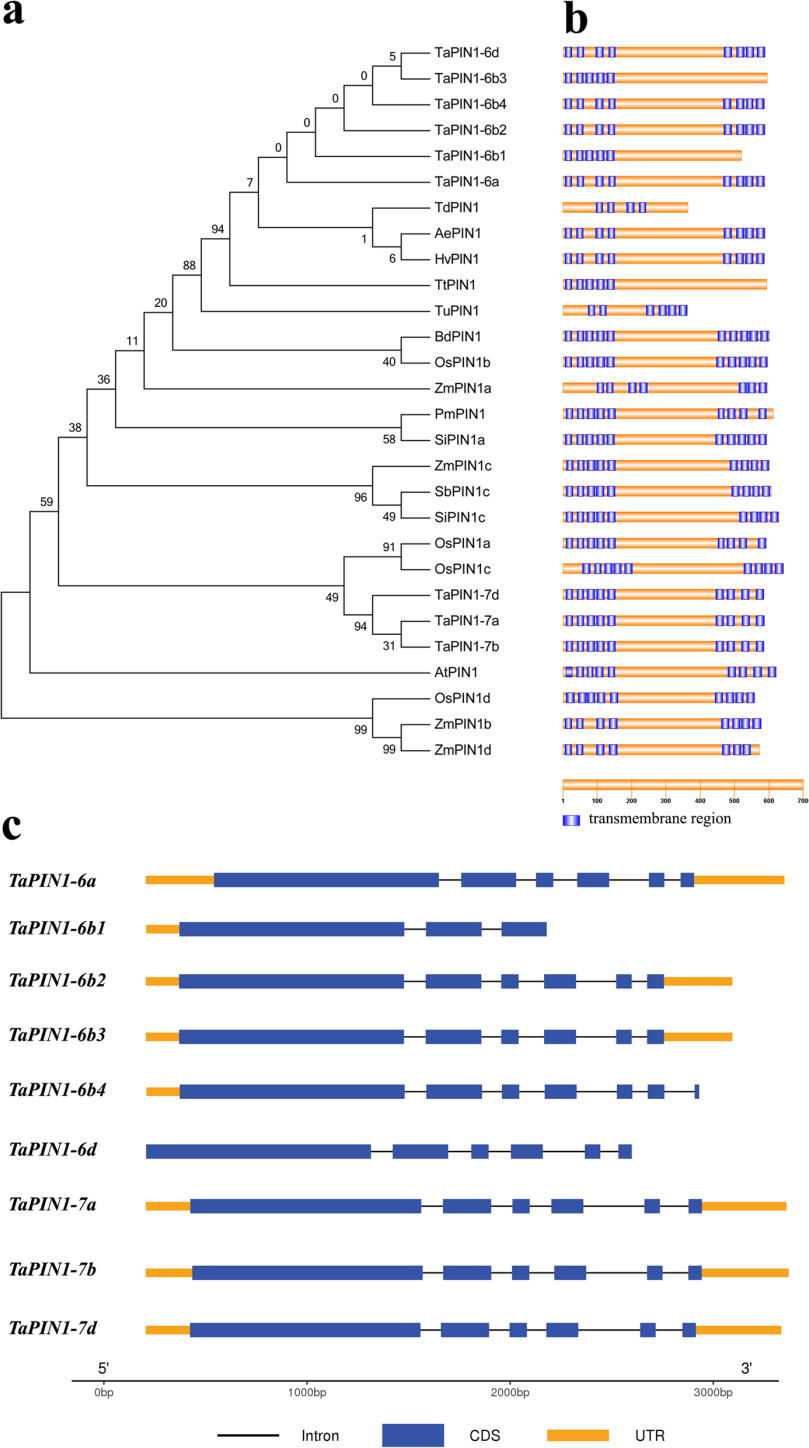
Phylogenetic analysis and predicted protein structure of PIN1s. **a** Phylogenetic analysis of PIN1 proteins in *A. tauschii* (Ae), *Arabidopsis thaliana* (At), *Brachypodium distachyon* (Bd), *H. vulgare* (Hv), *Oryza sativa* (Os), *Panicum miliaceum* (Pm), *Setaria italica* (Si), *Sorghum bicolor* (Sb), *T. aestivum* (Ta), *T. dicoccoides* (Td), *Triticum turgidum* subsp. *durum* (Tt), *Triticum urartu* (Tu), and *Zea mays* (Zm). **b** Predicted protein structure of PIN1s transporters. Blue squares show the predicted transmembrane domains of proteins. **c** Structural schematic of *TaPIN1* genes. Black line, blue squares, and yellow squares indicate intron, CDS, and UTR, respectively

The dendrogram showed that the TaPIN1-6 proteins were closely related to the PIN1 proteins in *Triticum dicoccoides*, *Aegilops tauschii*, and *Hordeum vulgare*, whereas the TaPIN1-7 proteins were closely related to the OsPIN1a and OsPIN1c proteins (Fig. 1a). By TMHMM2 [23] (http://www.cbs.dtu.dk/services/TMHMM-2.0/) analysis, TaPIN1 proteins harbor the transmembrane helices predicted as the trait of auxin transport carrier candidates (Fig. 1b). Consequently, each TaPIN1s had a typical structure. The distinct central hydrophilic loop of variable length separating two hydrophobic domains of about four transmembrane regions [11], which are similar to ZmPIN1s [24] and OsPIN1s proteins [25], except for TaPIN1-6b1 and TaPIN1-6b3, lacking transmembrane domains in their C-terminals. Additionally, we analyzed the exon–intron structure schematic of *TaPIN1* genes. All the *TaPIN1* genes contain six exons and five introns excepted *TaPIN1-6b1* gene (Fig. 1c), suggested that *PIN1* gene family is conservative.

### Expression patterns of TaPIN1 genes and subcellular localization of TaPIN1-6a protein

**Fig. 2:**
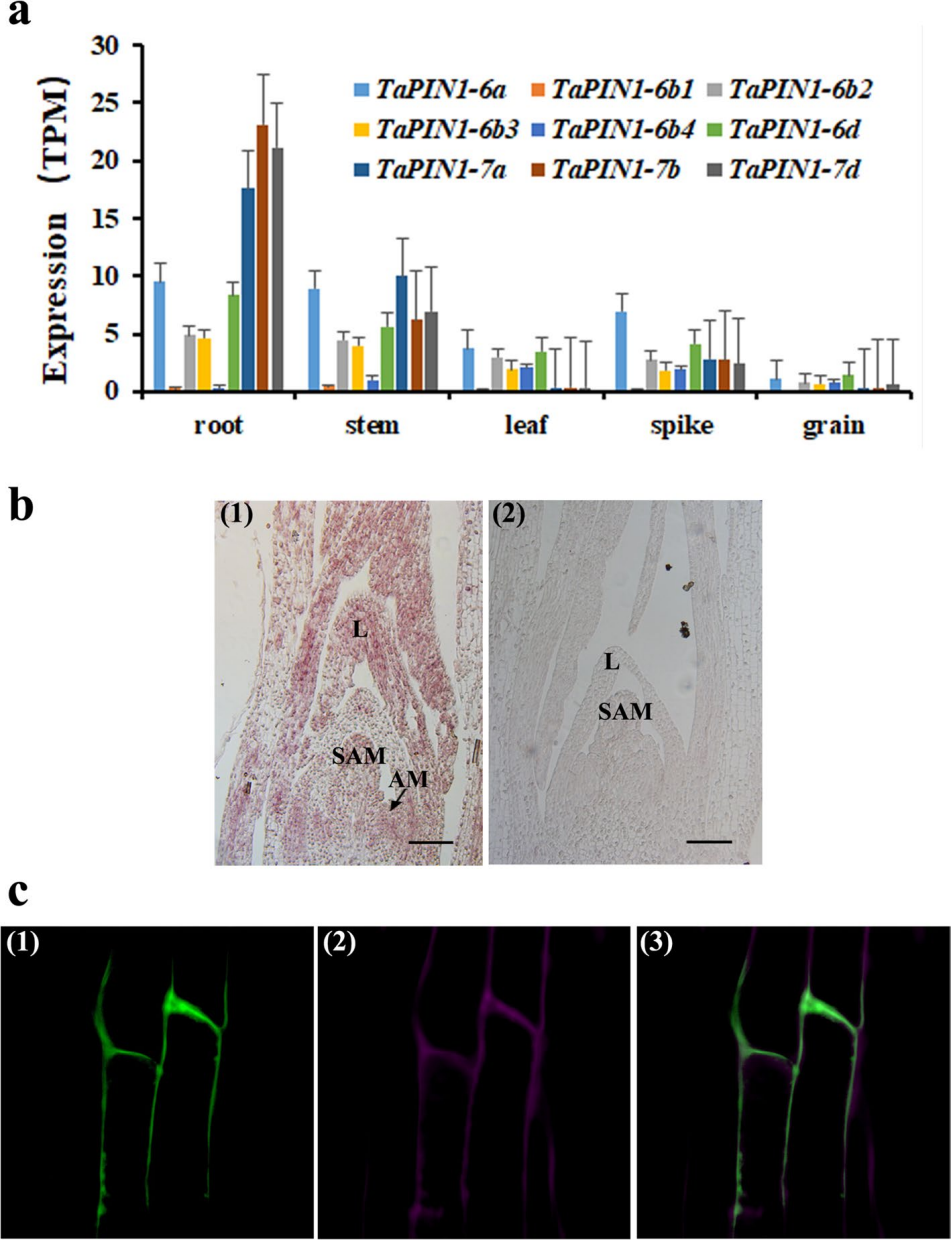
Expression patterns of *TaPIN1* genes and subcellular localization of TaPIN1-6a protein. **a** Normalized gene expression (TPM) analysis of the *TaPIN1* genes from five different tissues. **b** (1) In situ hybridization analysis of the expression of *TaPIN1-6* in the stem apex and young leaves of wheat. (2) The hybridization result of the sense probe. L: leaf. SAM: shoot apical meristem. AM: axillary meristem. Bars indicate 100 μm. **c** (1) Fluorescence signal in *Arabidopsis* root of *35S::TaPIN1-6a-CDS-GFP*. (2) FM4-64 staining. (3) Merged

Nine *TaPIN1* genes were expressed, but the expression level of each gene was different (Fig. 2a). In general, *TaPIN1* genes were abundant in the root and stem; the least expression level was observed in the grain. Among different *TaPIN1* genes, the expression abundance of *TaPIN1-6a*, *TaPIN1-6b2*, *TaPIN1-6b3*, and *TaPIN1-6d* was generally high in each tissue, particularly in the root, stem, and spike; moreover, the expression level of *TaPIN1-6a*, *TaPIN1-6d*, *TaPIN1-6b2*, and *TaPIN1-6b3* was from high to low. Additionally, *TaPIN1-6b1* had the lowest expression level in all tissues, and *TaPIN1-6b4* had lower expression level in the stem and root. *TaPIN1-7a*, *TaPIN1-7b*, and *TaPIN1-7d* had a high expression level in the root, high abundance in the stem and spike, and low expression level in the leaf and grain. In situ hybridization showed that six members of *TaPIN1-6* genes on chromosomes 6 were strongly expressed in the stem apex, axillary bud, and young leaf in the single ridge stage (Fig. 2b).

Based on expression pattern analysis of *TaPIN1* genes, *TaPIN1-6a* gene was generally high in each tissue. To explore the subcellular localization of the TaPIN1 proteins, *35S*::*TaPIN1-6a-CDS-GFP* fusion expression vector was constructed and infected into *Arabidopsis*. Strong fluorescence signals in the *TaPIN1-6a-CDS-GFP* transgenic plants were located on the plasma membrane in the root, thereby indicating that TaPIN1s are plasma membrane-localized proteins (Fig. 2c).

### Genetic transformation of wheat and molecular identification of transgenic plants

**Fig. 3:**
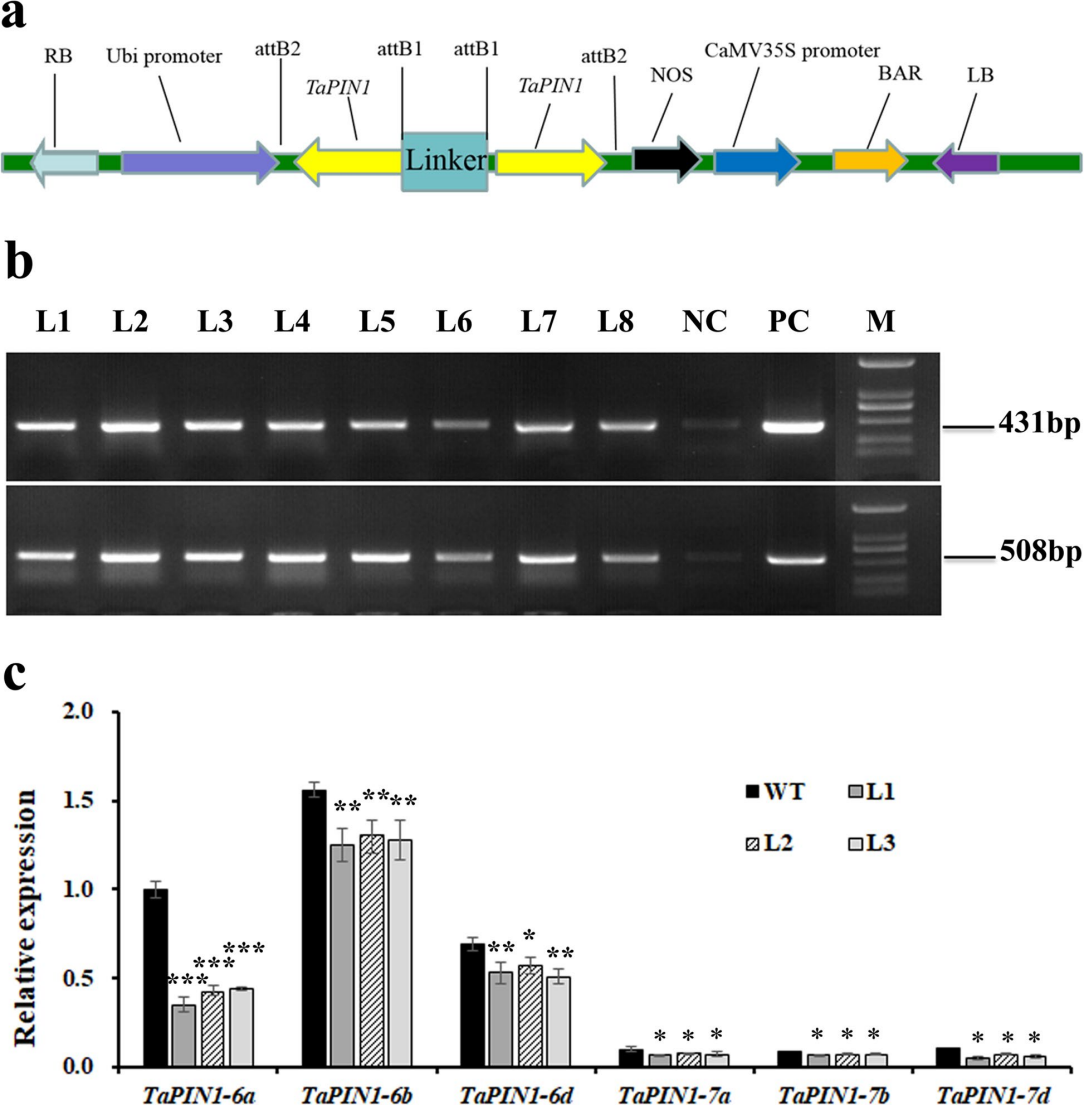
Genetic transformation of wheat and molecular identification of transgenic plants. **a**. The structure of the *TaPIN1-RNA* interference vector. RB: right boundary. LB: left boundary. **b**. Molecular identification of transgenic plants. (1) *bar* gene. (2) *TaPIN1* genes. L1-L8: *TaPIN1-RNAi* transgenic lines. NC: negative control. PC: positive control. M: 2000 bp marker. **c**. qRT-PCR analysis of *TaPIN1* genes expression. WT: wild type. L1-L3: transgenic lines 1–3 (*P < 0.05, **P < 0.01, ***P < 0.001)

*TaPIN1-RNA* interference vector (*TaPIN1-RNAi*) was constructed (Fig. 3a) and transformed into wheat (*T. aestivum* cv. CB037) by *Agrobacteria*-mediated genetic transformation to identify the function of *TaPIN1* genes in wheat development.

A total of 77 resistant plants were obtained, in which eight *TaPIN1-RNAi-*positive plants were obtained by polymerase chain reaction (PCR)-based identification. *Bar* (selective marker gene) and *TaPIN1* genes were confirmed simultaneously in transgenic lines (Fig. 3b). Three T_4_ generation plants, namely, L1, L2, and L3, were selected for further analysis. Quantitative real-time PCR (qRT-PCR) showed that the expression level of *TaPIN1* genes in axillary bud after gemination 20 days was significantly decreased in L1, L2, and L3 compared with that in the wild type, particularly the expression level of *TaPIN1-6a* (Fig. 3c).

### Knock down expression of TaPIN1 genes increased the tiller number of transgenic wheat lines

**Fig. 4:**
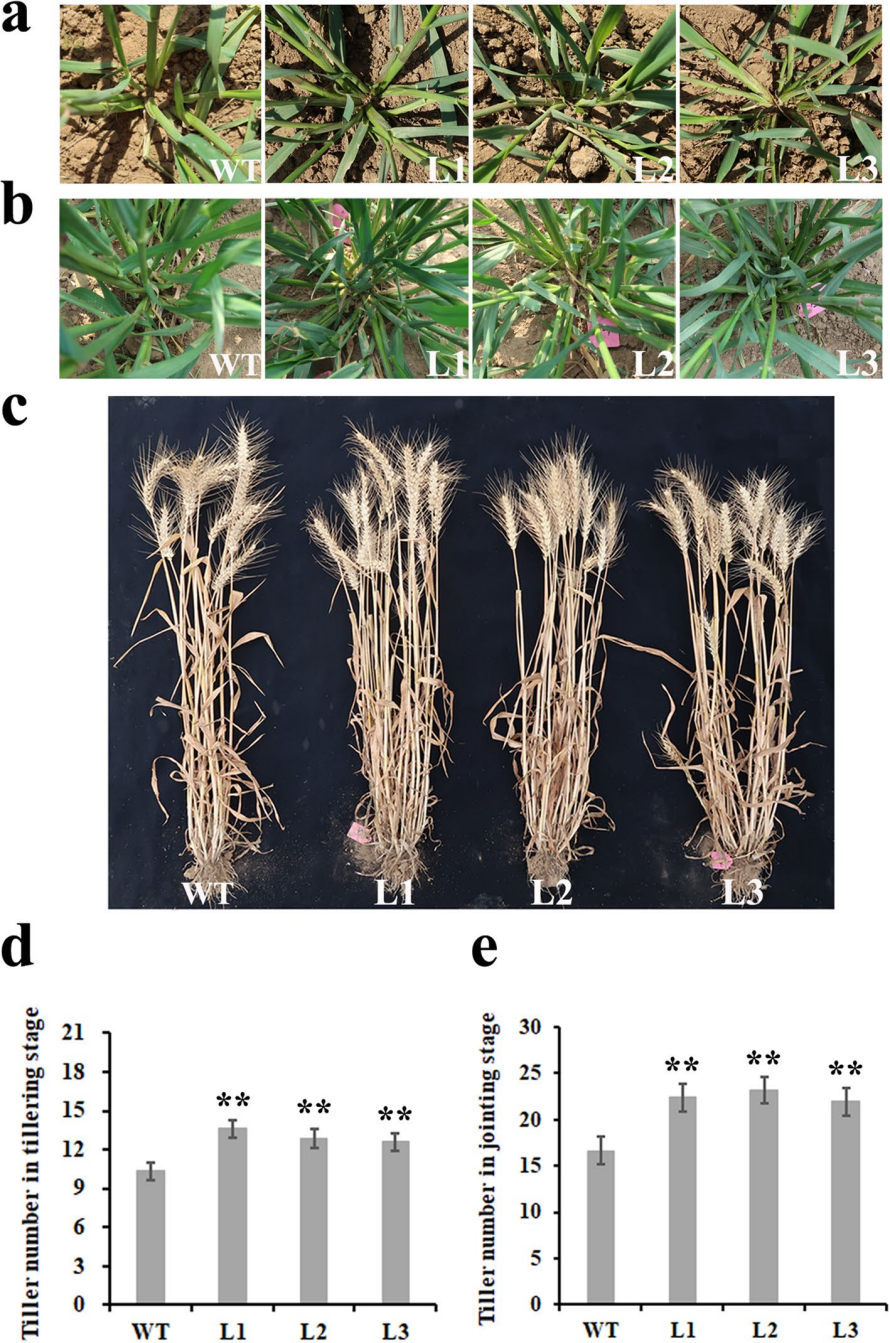
Increased tiller number of *TaPIN1-RNAi* transgenic lines in different developmental stages. **a** and **d**. tillering stage. **b** and **e**. jointing stage. **c**. mature stage. WT: wild type. L1-L3: transgenic lines 1–3 (*n* = 15, *P < 0.05, **P < 0.01)

Three transgenic lines, namely, L1, L2, and L3, and wild type (cv. CB037) were sown in field to observe the agronomic characters. Tiller number was analyzed at three developmental stages, that is, tillering stage (Fig. 4a), jointing stage (Fig. 4b), and mature stage (Fig. 4c). We found that the tiller number were increased significantly compared with those of the wild type in the tillering stage and jointing stage (Fig. 4d and e). These results suggest that *TaPIN1s* play a role in the regulation of tiller number.

### Increased productive tiller number and grain yield per plant in transgenic lines

**Fig. 5:**
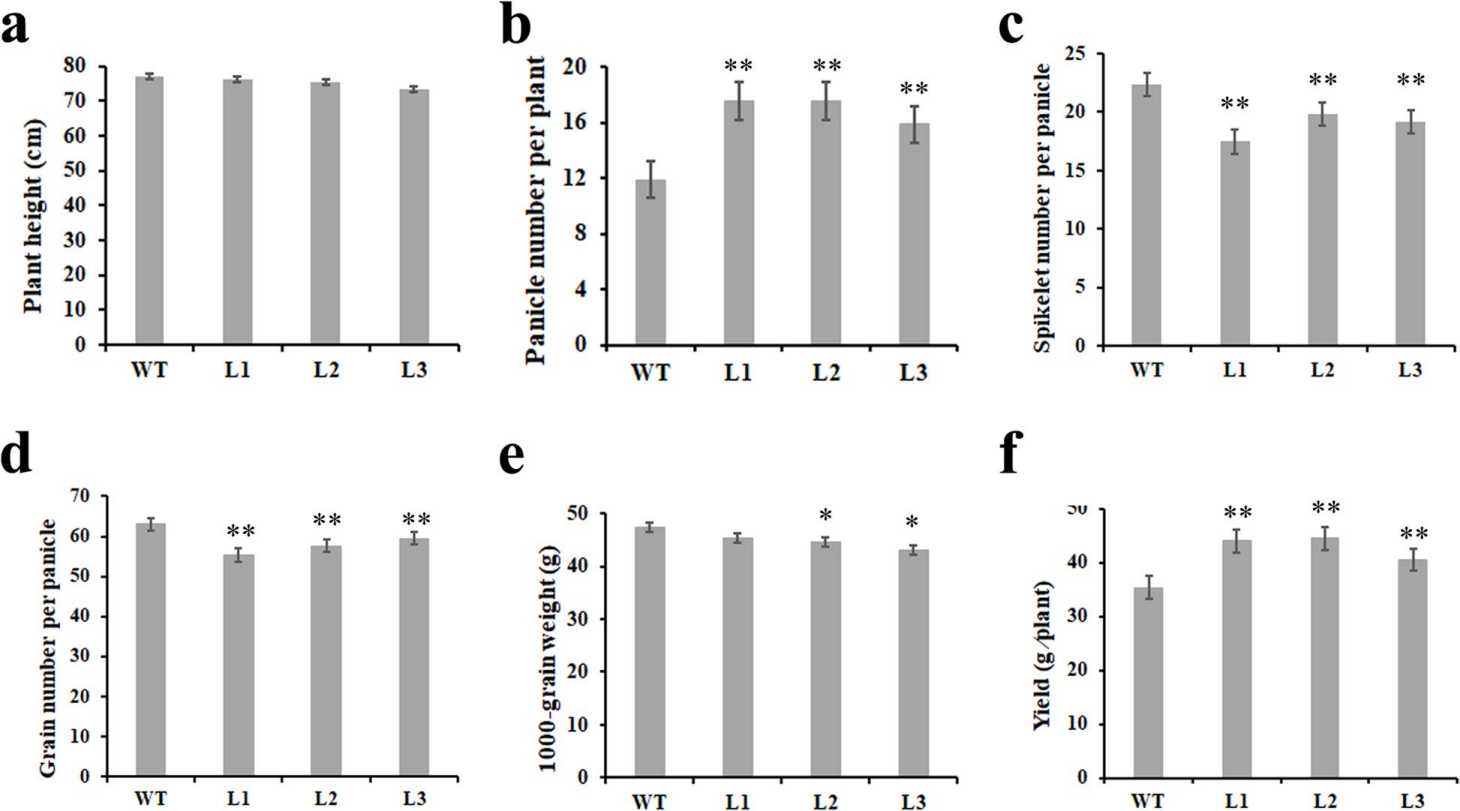
Agronomic traits of mature wheat in the *TaPIN1-RNAi* transgenic lines. **a**. plant height. **b**. panicle number per plant. **c**. spikelet number per panicle. **d**. grain number per panicle. **e**. 1000-grain weight. **f**. yield per plant. WT: wild type. L1-L3: transgenic lines 1–3 (*n* = 15, *P < 0.05, **P < 0.01)

The agronomic traits of the L1, L2, and L3 transgenic lines, such as plant height, productive tiller number, spikelet number per panicle, grain number per panicle, 1000-grain weight, and grain yield per plant, were evaluated after harvest. The plant height of the L1, L2, and L3 transgenic lines was decreased slightly compared with that of the wild type (Fig. 5a). The productive tiller number and grain yield per plant of three transgenic lines were increased compared with that of the wild type and reached a significant difference level (Fig. 5b and f). By contrast, spikelet number per panicle and grain number per panicle were significantly reduced compared with wild type (Fig. 5c and d). The 1000-grain weight also declined significantly in the L2 and L3 lines (Fig. 5e). Thus, a reduction expression of *TaPIN1s* increased the productive tiller number and grain yield per plant of wheat.

## Discussion

The optimization of plant architecture is an important goal for breeders to breed high-yielding wheat cultivars. As a key regulator of plant developmental processes, auxin plays roles in regulating the production of branch by mediating the meristem [7, 26–28]. The rice and maize *PIN1* gene family had four members, whereas the wheat *PIN1* family had nine members. Phylogenetic analysis suggested that six TaPIN1-6, TdPIN1, AePIN1, and HvPIN1 proteins and three TaPIN1-7, OsPIN1a, and OsPIN1c proteins formed a group separately (Fig. 1). In addition, the *TaPIN1* genes contained four copies in genome B of wheat, which might have functional redundancy and complementarity. However, the potential mechanism of multiple members of TaPIN1s and different copies of TaPIN1-6b remains to be investigated.

In maize, *ZmPIN1* marks the L1 layer of the shoot apical meristem and inflorescence meristem during the flowering transition, and ZmPIN1-mediated auxin transport is related to cellular differentiation during maize embryogenesis and endosperm development [29]. The high transcript levels of *OsPIN1a* and *OsPIN1b* were observed in the root, stem base, stem, leaf, and young panicle, whereas a relatively low level of *OsPIN1c* was observed in the leaf and young panicle of rice [25]. We found that *TaPIN1-6* genes were strongly expressed in the stem apex and young leaf in the single ridge stage of wheat. The PIN1 protein belongs to a subfamily that has a “long” central hydrophilic loop and hydrophobic domain. The hydrophobic domain sequence of PIN1s, primarily in the transmembrane helices, is highly conserved, and it does not tolerate insertions or deletions [11]. Nine candidate members of the TaPIN1 proteins harbor the transmembrane regions predicated by TMHMM2 [23], and TaPIN1s has the typical structure except for TaPIN1-6b1 and TaPIN1-6b3, which do not possess the predicated transmembrane region in C-terminal (Fig. 1b). However, the transmembrane region in the hydrophobic domain is important for PIN1 function, lack of which may results in loss of function in plants. Based on previous studies, no PIN1 protein lacks the transmembrane region in maize and rice at the C-terminal; thus, the function of TaPIN1-6b1 and TaPIN1-6b3 needs to be further investigated.

Endogenous hormones affect shoot branching [3, 7, 30]. Tiller number in monocots, such as wheat and rice, is closely associated with yield. Excessive tiller in cereal crop can lead to yield reductions because tillers compete for resources, and many secondary tillers are not fertile [31]. *PIN1* genes play roles in the regulation of tiller number and tiller angle in rice [21] and switchgrass [17]. In this study, we found that the down-expression of *TaPIN1s* significantly increased tiller number at the tillering stage, jointing stage, and mature stage in wheat. These phenotypes are similar to those of rice and switchgrass with down-expression of *PIN1* [21]. In addition, we found that the down-expression of *TaPIN1s* reduced the spikelet number per panicle, grain number per panicle, and the 1000-grain weight, but increased the productive tiller number and grain yield per plant. Thus, *TaPIN1s* function in lateral bud initiation and increase the yield per plant in wheat.

It was reported that virus-induced gene silencing of the *TaPIN1* genes resulted in 26% reduction in plant height [32]. Multiple sequence alignment revealed that the *TaPIN1* genes in the study by Singh et al. [32] were similar to TaPIN1-7b in the phylogenetic analysis of our study (Fig. 1a). However, no obvious change in plant height was observed in the *TaPIN1-RNAi* transgenic lines in our study. We suggest that *TaPIN1s* in different chromosomes may have subfunctionalization and play different roles in wheat. In addition, different transformation strategies, virus-induced gene silencing, and the *Agrobacterium*-mediated method may lead to a difference in the expression level of *TaPIN1* genes in transgenic plants, which may improve plant height.

## Conclusions

In this study, we identified nine homologs of *PIN1* genes in wheat, of which *TaPIN1-6* genes showed higher expression in the stem apex and young leaf in wheat, and the TaPIN1-6a protein was localized in the plasma membrane. The down-expression of *TaPIN1s* increased the tiller number in *TaPIN1-RNAi* transgenic wheat plants, indicating that auxin might mediate the axillary bud production. By contrast, the spikelet number, grain number per panicle, and the 1000-grain weight were decreased in the *TaPIN1-RNAi* transgenic wheat plants compared with those in the wild type. Our study suggests that *TaPIN1s* is required for the regulation of grain yield in wheat.

## Methods

### Phylogenetic analysis and protein structure prediction

PIN proteins were aligned with MEGA6.06, and a maximum likelihood phylogenetic tree was prepared with Phylip package using the 500-bootstrap method. The transmembrane helices of TaPIN1 proteins were predicted using TMHMM2 [23] (http://www.cbs.dtu.dk/services/TMHMM-2.0/).

### Subcellular localization and confocal microscopy

*35S*::*TaPIN1-6a-CDS-GFP* was inserted into the pMDC83 vector and infected into *Arabidopsis* by *Agrobacterium tumefaciens* (strain GV3101)-mediated transformation to determine the subcellular localization of TaPIN1. Then, T_1_ transgenic plants were placed in hygromycin (15 mg/L) pressure medium, and after germination, 5 to 6-day-old seedling roots were excised for imaging as described [33]. Staining of roots with FM4-64 was performed as previously described [34–39]. Confocal microscopy was performed on the root of the positive *Arabidopsis* plants with a Leica TCS SP5II (Richmond, IL, USA), and the GFP signal was observed at 505 to 530 nm emission under 488 nm excitation. Fluorescent images were captured using an LSM 880 Airyscan (Zeiss, German) with a 40 × objective. Fluorescence was detected using a 488-nmbandpass filter for GFP. Images were processed using LSM image processing software (Zeiss, German).

### In situ hybridization

*In situ* hybridization and detection of hybridized signals were carried out as described by Meng et al. [40]. The wheat stem apex was fixed in 4% v/v paraformaldehyde (Sigma, USA) and 0.1 M phosphate buffer (pH 7.0) overnight at 4 °C. Then, the specimens were embedded in paraplast (Sigma, USA) and sectioned at 8.0 μm. Antisense and sense RNA probes were synthesized using a digoxigenin RNA labeling kit (Sigma-Aldrich, USA). The antisense RNA probe for *TaPIN1-6* genes was amplified by PCR using the upper primer 5′- ACCGGACTACAACGACGCG—3′ and lower primer 5′—GAATTGTAATACGACTCACTATAGGGGCGCCCATCCCATTGTTGTT—3′. The sense RNA probe was amplified by PCR using the upper primer 5′- GAATTGTAATACGACTCACTATAGGGACCGGACTACAACGACGCG—3′ and lower primer 5′—GCGCCCATCCCATTGTTGTT—3′.

### Gene expression pattern analysis

Expression pattern analysis of the *TaPIN1* genes was performed on the expVIP, which was developed by Ricardo H. Ramirez-Gonzalez (github) and Bijan Ghasemi Afshar (github) as part of the Designing Future Wheat Institute Strategic Program within the Uauy lab (http://www.wheat-expression.com/) [41, 42]. The numbers of *TaPIN1* genes in wheat *EnsemblPlants* database were used as a query for the analysis of the expVIP.

### Plasmid construction and wheat transformation

For the RNA-interfering plasmid construct, a 242 bp cDNA fragment between 1 and 242 of the *TaPIN1s* cDNA was amplified by PCR using the upper primer 5′- CACCATGATCACGGGCACGGACTTCT—3′ and lower primer 5′—ATGAGCTTCTGCAGCGTGTCG—3′. The PCR product was inserted into the RNAi vector PC336 to trigger specific RNAi of *TaPIN1s* in wheat, which created a construct, namely, *TaPIN1-RNAi*. Then, the construct was transformed into callus initiated from immature embryo of CB037 via *A. tumefaciens* (strain C58C1)-mediated transformation [43]. Strain C58C1 was kindly provided by Dr. Tom Clemente at the University of Nebraska-Lincoln, USA. The PC336 vector was kindly provided by Dr. Daolin Fu at the Department of Plant, Soil and Entomological Sciences, University of Idaho, Moscow, Idaho, USA.

### Molecular identification and qRT-PCR analysis

Genomic DNA was isolated from the putative transgenic wheat plants following the CTAB method [44]. In addition, PCR was used to verify the candidate transgenic plants using the gene-specific upper primer 5′- TTTTAGCCCTGCCTTCATACG—3′ and lower primer 5′—ATGAGCTTCTGCAGCGTGTCG—3′.

Ultrapure RNA Kit (CoWin Bio., China) was used for total RNA extraction according to the manufacturer’s instruction in our experiment, and the transcript levels of *TaPIN1* genes in transgenic wheat lines were analyzed by qRT-PCR (Roche Real-Time quantitative PCR, Roche). The first-strand cDNAs were synthesized by using Transcript One-Step gDNA Removal and cDNA Synthesis Supermix (TransGen Biotech Co., LTD, China). qRT-PCR was performed using the gene-specific primers in Table 1 as described by Zhao et al. [45]. The experiments were independently replicated three times under the same conditions.

**Table 1:** Primers used for qRT-PCR

Primer name	sequence
Taactin-F	5′-AGTCGAGAACGATACCAGTAGTACGA-3′
Taactin-R	5′-GCCATGTACGTCGCAATTCA-3′
TaPIN1-6a-F	5′-TCATGGTGCAGATCGTCGTC-3′
TaPIN1-6a-R	5′-CGGTGATGAGCATGCGGGC-3′
TaPIN1-6b-F	5′-CAATCGAGACCGAGGCC-3′
TaPIN1-6b-R	5′-GCGTTGGTGAGGTTGCTGG-3′
TaPIN1-6d-F	5′-GGCGGACCCGAACAACAATG-3′
TaPIN1-6d-R	5′-TGAGGCTGGAGTAGGTGTTG-3′
TaPIN1-7a-F	5′-GCCGGCAACAACAACAACAAC-3′
TaPIN1-7a-R	5′-GAGCTCCACACGAACATGTG-3′
TaPIN1-7b-F	5′-GAGACGGAGGCGGAGGTC-3′
TaPIN1-7b-R	5′-CATGGAGCGGCGCGAGTAT-3′
TaPIN1-7d-F	5′-GAGGACAAGGCCGGCGG-3′
TaPIN1-7d-R	5′-GAGCTCCACACGAACATGTG-3′

### Plant growth conditions

Wild type and T_4_ transgenic plants were grown at the Experimental Station of Shandong Agricultural University, Tai’an, Shandong Province, China. Wheat grains were sown and covered with plastic sheeting for insulation on November 17, 2018. Plastic sheeting was removed on March 15, 2019 after 5 days for ventilation and harvested on June 12, 2019. Each experimental line was sown with 25 cm in-row spacing and 10 cm plant-to-plant spacing. Common wheat cv. CB037 was kindly provided by Prof. Xiao Chen at the Institute of Crop Science of the Chinese Academy of Agricultural Sciences in Beijing, China. *Arabidopsis* for subcellular localization was an ecotype of Columbia-0 (Col.-0), which was planted in the growth room in the Science and Technology Innovation Building of Shandong Agricultural University. The growth room was set at 22 °C and 16 h day-time/8 h night-time mode. Moreover, IBM SPSS Statistics was used in our study for statistical analysis of data.

## Data Availability

All data generated or analyzed in this study are included in this published article. The datasets used and/or analyzed during the current study are available from the corresponding author on reasonable request. The protein sequences of *T. aestivum* (Ta) were downloaded from http://plants.ensembl.org/Triticum_aestivum/Info/Index, and the protein sequences of *A. tauschii* (Ae), *A. thaliana* (At), *B. distachyon* (Bd), *H. vulgare* (Hv), *O. sativa* L. (Os), *P. miliaceum* L. (Pm), *S. italica* (Si), *S. bicolor* (Sb), *T. dicoccoides* (Td), *T. turgidum* subsp. *durum* (Tt), *T. urartu* (Tu), and *Z. mays* (Zm) were downloaded from https://www.ncbi.nlm.nih.gov/. The accession number of the PIN1 proteins are as follows: TaPIN1-6a (TraesCS6A02G308600.1), TaPIN1-6b1 (TraesCS6B02G337300.1), TaPIN1-6b2 (TraesCS6B02G337300.2), TaPIN1-6b3 (TraesCS6B02G337300.3), TaPIN1-6b4 (TraesCS6B02G337300.4), TaPIN1-6d (TraesCS6D02G287800.1), TaPIN1-7a (TraesCS7A02G190600.1), TaPIN1-7b (TraesCS7B02G095500.1), TaPIN1-7d (TraesCS7D02G191600.1), AePIN1 (XP_020171849.1), AtPIN1 (NP_177500.1), BdPIN1 (XP_003570666.2), HvPIN1 (BAJ97950.1), OsPIN1a (AGV28593.1), OsPIN1b (AGV28594.1), OsPIN1c (XP_015641301.2), OsPIN1d (XP_015619425.1), PmPIN1 (RLM78735.1), SbPIN1c (XP_002436761.1), SiPIN1a (XP_004953880.1), SiPIN1c (XP_022681920.1), TdPIN1 (TRIDC6AG046430.1), TtPIN1 (VAI60845.1), TuPIN1 (TRIUR3_21048), ZmPIN1a (PWZ26735.1), ZmPIN1b (PWZ29332.1), ZmPIN1c (NP_001309394.1), and ZmPIN1d (AQK57225.1).
